# Meditation acutely improves psychomotor vigilance, and may decrease sleep need

**DOI:** 10.1186/1744-9081-6-47

**Published:** 2010-07-29

**Authors:** Prashant Kaul, Jason Passafiume, Craig R Sargent, Bruce F O'Hara

**Affiliations:** 1 Department of Biology, University of Kentucky, Lexington, KY, USA; 2 Psychiatry and Behavioral Sciences, SUNY Upstate Medical Center, Syracuse, NY, USA

## Abstract

**Background:**

A number of benefits from meditation have been claimed by those who practice various traditions, but few have been well tested in scientifically controlled studies. Among these claims are improved performance and decreased sleep need. Therefore, in these studies we assess whether meditation leads to an immediate performance improvement on a well validated psychomotor vigilance task (PVT), and second, whether longer bouts of meditation may alter sleep need.

**Methods:**

The primary study assessed PVT reaction times before and after 40 minute periods of mediation, nap, or a control activity using a within subject cross-over design.

This study utilized novice meditators who were current university students (n = 10). Novice meditators completed 40 minutes of meditation, nap, or control activities on six different days (two separate days for each condition), plus one night of total sleep deprivation on a different night, followed by 40 minutes of meditation.

A second study examined sleep times in long term experienced meditators (n = 7) vs. non-meditators (n = 23). Experienced meditators and controls were age and sex matched and living in the Delhi region of India at the time of the study. Both groups continued their normal activities while monitoring their sleep and meditation times.

**Results:**

Novice meditators were tested on the PVT before each activity, 10 minutes after each activity and one hour later. All ten novice meditators improved their PVT reaction times immediately following periods of meditation, and all but one got worse immediately following naps. Sleep deprivation produced a slower baseline reaction time (RT) on the PVT that still improved significantly following a period of meditation. In experiments with long-term experienced meditators, sleep duration was measured using both sleep journals and actigraphy. Sleep duration in these subjects was lower than control non-meditators and general population norms, with no apparent decrements in PVT scores.

**Conclusions:**

These results suggest that meditation provides at least a short-term performance improvement even in novice meditators. In long term meditators, multiple hours spent in meditation are associated with a significant decrease in total sleep time when compared with age and sex matched controls who did not meditate. Whether meditation can actually replace a portion of sleep or pay-off sleep debt is under further investigation.

## Background

Meditative practices have gained increasing attention in the West over the past several decades. Previous studies on meditation have documented clear changes in the EEG that are distinct from sleep and typical wake, as well as a variety of other physiological changes [1-5]. The most dramatic and immediate change in the EEG is the dominance of alpha waves (8-12 Hz) across much of the cortex [3,6]. Although this ordinarily occurs with simple eyes-closed resting behavior, the magnitude of this change is greater during meditation. Furthermore, theta bursts appear more commonly during meditation. While meditation and eyes closed resting are not a state of sleep, the dominance of alpha waves has some superficial similarities to the dominance of delta waves (0.5-4 Hz) that occurs during deep non-REM sleep (relatively high voltage, synchronous waves). While a dominance of alpha or delta waves are generated by very different neural systems, they both reflect, ultimately, an increased synchronous firing pattern in cortical neurons as measured by the EEG [7,8]. The benefit (if any) of neurons firing together is unclear, but it may provide a time to re-set or optimize the brain for new tasks. Surprisingly, given the number of EEG studies on meditation, essentially no one has addressed whether meditation might provide a restorative function similar to non-REM sleep despite centuries of anecdotal claims by certain practitioners and followers that meditation does reduce the need for sleep [1,3,9]. In contrast, several studies have addressed whether meditation may affect the quality of subsequent sleep, and whether meditation may help those with insomnia fall asleep more easily [9-15].

A major difficulty in assessing whether meditation may be able to replace a portion of sleep, is that the functions of sleep are not well understood, and no direct measure of sleep-need exists. However, several indirect measures are available including Multiple Sleep Latency Tests (MSLT), Maintenance of Wakefulness Tests (MWT), EEG variables such as delta power during non-REM sleep, or performance measures such as the Psychomotor Vigilance Task (PVT). Performance on virtually all tests declines with decreasing amounts of sleep, ranging from driving skills to simple reaction time [16]. The PVT, as used in this study, requires the subject to monitor a LED display and hit a button as soon as it starts counting, and to respond repeatedly at random intervals over a ten minute time period. Typical response times range from about 180 msec to 300 msec in well rested subjects, with almost no lapses, while reaction times and lapses increase with increasing sleep debt [17].

As a first step, we began with a simpler question, asking whether meditation could provide an immediate improvement in PVT performances under conditions of very mild (normal afternoon levels) and severe sleep debt (no sleep for one night), respectively. We compared the same subjects following forty minute bouts of meditation, nap, or control activity on multiple days. Since forty minute bouts of meditation are probably not sufficient to substantially affect sleep debt, we also investigated typical sleep duration and other variables in long-term meditators who meditate 2-3 hours or more per day. This latter study may help address whether meditation (or even simple eyes-closed resting) can be used to pay-off some portion of sleep debt, either through the neuronal synchronization seen by EEG, or through some other mechanism.

## Methods

In the first study on novice meditators, participants included 7 males and 3 females - paid volunteers 19 to 23 years old (5 Caucasian, 3 Asian and 2 Hispanic). Average age was 21.5 years. Subjects were recruited by campus advertisements at the University of Kentucky or word of mouth. All ten accepted subjects appeared in excellent health, and underwent general screening using a detailed questionnaire to eliminate those with medical or psychiatric illness or sleep disorders, and limited use of caffeine, alcohol, and other drugs. Two subjects were screened out and did not participate based on this information. Subjects were instructed to abstain from caffeine, nicotine, alcohol, and all other drugs on each study day. They were also instructed to keep a regular sleep-wake schedule, for a week prior to each testing day. The sleep-wake behavior was monitored in all subjects prior to each experimental day using an activity monitor watch (IM Systems, ACTITRAC) to ensure conformity. Average sleep time was 7.48 ± 0.40 hours/day on the nights prior to each experimental session (based on actigraphy data), which was also generally consistent throughout the experimental period. Subjects typically had late bed-times (around 1:00 am) and late wake-times (around 8:30 am), as is common in a young university community.

Subjects were tested under four treatments: Control (C), Nap (N), Meditation (M) and Sleep Deprivation plus Meditation (SD+M). For the first three treatment conditions, C, N and M, each subject was tested twice (a total of 6 different days), and once for the SD+M treatment. The testing was done on non-consecutive days, and the activity for each day was randomly assigned (without the subjects' prior knowledge), with the one night of total SD completed over a weekend. Each subject refrained from caffeine and other stimulants or depressants throughout each experiment. Each subject was given 2 practice runs on the PVT, ahead of the experimental days. None of the subjects had prior experience with meditative techniques. Each subject was given instructions in simple eyes-closed concentrative meditation techniques (with focus on breathing) for 2 days in pairs (one hour per day), ahead of the experimental schedule. All training was done by the same individual who practices such meditation and had recently completed a twelve-week course in order to provide more uniform instruction. Subjects were taught in the kneeling position with the aid of a kneeling meditation bench (Samadhi Cushions, Barnet, Vermont, USA) that is especially helpful for beginners in optimizing spinal alignment and reducing weight and stress on the knees, hips, ankles, and back. The bench was covered with the optional cushion, along with a floor cushion, for further comfort. Subjects were taught uniform abdominal breathing, with focus on abdominal movements throughout the 40 minute meditation period. On control days, subjects sat in a standard desk chair, listened to soft music and engaged in either light reading or conversation to ensure a constant period of eyes open wakefulness. On nap days, the subjects were asked to lie down in bed and attempt to sleep for the entire 40 minutes. All conditions were completed in a quiet room of the subjects' choosing.

PVT-192 (Ambulatory Monitoring Inc.) was used to test for vigilance and reaction time. Three 10 min tests were done at 3:00 pm, 4:00 pm and 4:50 pm respectively. The treatments(C, N, and M) took place between 3:10 pm and 3:50 pm. Between 4:10 pm and 4:50 pm, each subject was involved in control activities the same as the control condition above (sitting, with light music background, reading or conversation, eyes open).

In the second study with long-term meditators, 7 subjects (3 females and 4 males, from India) with at least 3 years of regular meditation practice (2 hrs or more per day for most days of the year) were used. Age range 24-48 years (all citizens of India in the Delhi region) and average age 38.1 years. All were healthy with no history of major medical, psychiatric or sleep problems. All practiced traditional yogic styles of meditation with focus on the breathing, and all would probably be classified as "concentrative" meditation as opposed to "mindfulness" meditation, although these distinctions are not always clear. Sleep journals were kept on a pre-supplied format for a minimum of 15 days (a maximum of 30 days). Activity monitors (ACTITRAC) were used for Actigraphy records for a minimum of 15 days to a maximum of 22 days. A marker button (read digitally) was pressed by the subjects every time they would commence to meditate. EEGs were done on a subset of subjects (n = 3) using a Neurocare Wingraph Digital EEG system (Biotech). A standard 10 lead placement system was used. MSLT and PVT tests were also conducted on a subset of subjects (n = 4) using standard methods. EEGs were scored by hand with the assistance of a trained and certified polysomnographic technician. Twenty-three control subjects in India were also selected for total sleep time comparisons relative to the seven meditators. These control subjects were sex and age matched.

All data were analyzed with analysis of variance (ANOVA) by using the General Linear Model (GLM) within SYSTAT 12 (SYSTAT Software, Inc., 1735, Technology Drive, Ste 430, San Jose, CA 95110, USA), or nonparametric alternatives when the assumptions of ANOVA were not met. We used the Kolmogorov-Smirnov, Shapiro-Wilk and Anderson-Darling tests for normality, and Levene's test for homogeneity of variances.

The first experiment was analyzed as a repeated measures design. The response variable was change in PVT reaction time, post- minus pre-. Each of 10 subjects was tested in each of 4 experimental treatments (Control, Nap, Meditation, Sleep Deprivation plus Meditation) in a two-way mixed model without replication, where Treatment was a fixed effect and Subject, the blocking variable, was a random effect. We were interested primarily in the treatment effects. The 4 treatments were compared with each other with a series of post hoc contrasts, and the resulting probabilities were Bonferonni adjusted. These 4 treatments were explored further with a repeated measures analysis of covariance (ANCOVA), where post- was our response variable, and pre- was our covariate.

**Figure 1:**
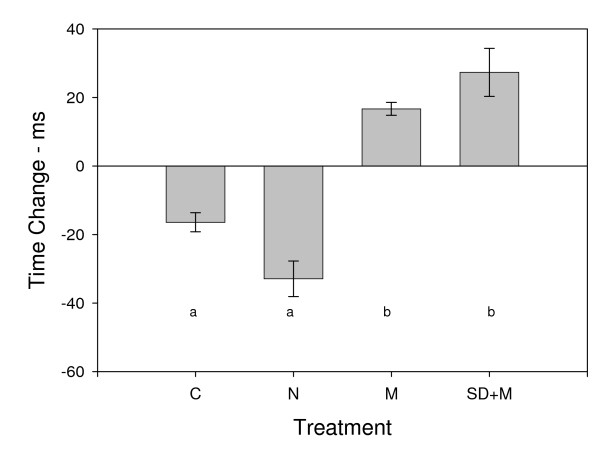
**Mean change in PVT reaction time for our four experimental treatments: Control (C), Nap (N), Meditation (M), Sleep Deprivation Plus Meditation (SD+M)**. Performance improves following meditation (M, SD+M) and declines following a nap and in controls (N, C); *Friedman Test Statistic = 23.4, df = 3, p << 0.0005*. Treatments noted with the same letter (a or b) denote nonsignificant subsets of the overall analysis. Values represent the mean PVT reaction times before treatment minus the post-treatment. Error bars denote one standard error.

We intended to analyze this response variable with a repeated measures analysis of variance (ANOVA), which is a two-way, randomized-block design, without replication. The two factors in the ANOVA are Treatment (fixed effect) and Subject (random effect, the blocking variable). Although our response variable satisfied the assumption of normality, it did not satisfy the assumption of homogeneity of variances (*Levene's test statistic = 5.890, p < 0.005*), and standard data transformations did not correct this problem. Variance heterogeneity presents problems for ANOVA. We rank-transformed the data, and found that the variances of the ranks among treatment groups were homogeneous (*Levene's test statistic = 1.769, p > 0.176*) and these ranks also satisfied the assumption of normality. We analyzed these ranks with the nonparametric alternative to a randomized block ANOVA, Friedman's Randomized Blocks. We found significant treatment effects on change in PVT reaction time (*Friedman Test Statistic = 23.4, df = 3, p << 0.0005; *Figure 1, Table 1). The M and SD+M treatments showed faster post-treatment reaction times, whereas the N and C treatments showed slower post-treatment reaction times (Figure 1, Table 1). We performed three post-hoc comparisons, the two treatments that had faster reaction times (M versus SD+M), the two treatments that had slower post-treatment reaction times (C versus N), and the treatments with meditation against those without meditation ([M plus SD+M] versus [C plus N]). Although these contrasts are orthogonal, they were unplanned. To preserve an experiment-wise error rate of 0.05, we applied the Bonferroni correction for these three contrasts, and compared their test statistics against *p < 0.0167*. These post hoc contrasts revealed that M and SD+M are one nonsignificant subset of the data (*Friedman Test Statistic = 1.6, df = 1, p > 0.20*), that C and N are another nonsignificant subset of the data (*Friedman Test Statistic = 3.6, df = 1, p > 0.05*), and that these two groups significantly differ from each other (*Friedman Test Statistic = 20.0, df = 1, P < 0.0005*). Note that doing a parametric ANOVA on the rank transformed data gives qualitatively the same results as the nonparametric Friedman's Randomized Blocks (Overall ANOVA: *F_3,27 _= 25.799, p << 0.0001*; M versus SD+M: *F_1,27 _= 0.893, p > 0.893*; C versus N: *F_1,27 _= 3.338, p > 0.078*; [M plus SD+M] versus [C plus N]: *F_1,27 _= 73.164, p << 0.0001*).

**Table 1:** The data on which Figures 1 and 2 are based. For the Nap, Control, and Meditation Treatments, the data are the averages for the 2 days of measurement.

	Control			Nap			Meditation			Sleep Deprivation + Meditation		
**Subject**	**Pre**	**Post**	**Diff**	**Pre**	**Post**	**Diff**	**Pre**	**Post**	**Diff**	**Pre**	**Post**	**Diff**

A	261.0	290.0	-29.0	287.5	312.0	-24.5	271.0	248.5	22.5	347.0	300.0	47.0
B	260.0	272.5	-12.5	237.5	290.5	-53.0	251.5	234.5	17.0	285.0	266.0	19.0
C	269.5	287.5	-18.0	284.5	328.0	-43.5	256.0	238.5	17.5	320.0	267.0	53.0
D	213.5	226.0	-12.5	253.5	310.0	-56.5	235.0	224.0	11.0	279.0	255.0	24.0
E	268.5	285.5	-17.0	221.5	252.0	-30.5	226.5	215.0	11.5	263.0	239.0	24.0
F	245.5	245.0	0.5	288.5	327.0	-38.5	287.5	262.0	25.5	275.0	288.0	-13.0
G	249.5	281.5	-32.0	246.5	239.5	7.0	243.0	225.0	18.0	234.0	219.0	15.0
H	268.0	292.0	-24.0	278.0	302.5	-24.5	273.0	252.5	20.5	334.0	282.0	52.0
I	239.0	249.0	-10.0	280.0	333.5	-53.5	251.5	241.0	10.5	299.0	252.0	47.0
J	223.0	233.0	-10.0	248.5	260.5	-12.0	238.5	226.0	12.5	234.0	229.0	5.0

**Figure 2:**
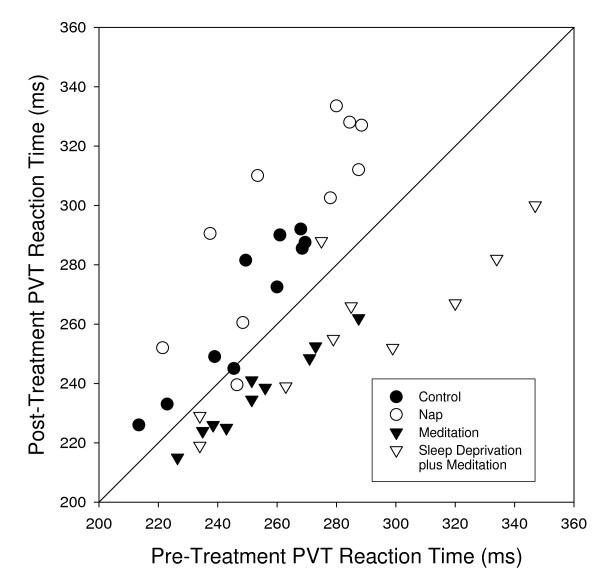
**A plot of Post-Treatment PVT Reaction Time versus Pre-Treatment PVT Reaction Time for our four treatments**. The treatments that included meditation, M and SD+M, showed faster post- than pre-treatment reaction times; whereas, the reverse was true for the C and N treatments. The M and SD+M treatments have a shallower regression slope than the two treatments that do not include meditation, C and N (*F_1,27 _= 12.610, p < 0.002*).

Unlike change in PVT reaction time, we found that pre- and post-treatment PVT reaction times do satisfy the assumptions of normality and homogeneity of variances. Therefore, we also performed a repeated measures analysis of covariance (ANCOVA) without replication, where post-treatment PVT reaction time was our response variable, our two factors were Treatment (fixed variable) and Subject (random blocking variable), and our covariate was pre-treatment reaction time. Although neither main factor was significant (Treatment: *F_3,23 _= 1.798, p = 0.176*; Subject: *F_9,23 _= 0.535; P = 0.834*), both the Covariate (Pre-: *F_1,23 _= 23.315. p <<0.0001*) and the Treatment By Covariate interaction term were significant (Treatment × Pre: *F_3,23 _= 3.13, p < 0.05*; the Subject by Covariate interaction term was not significant, *F_9,14 _= 0.318. p = 0.955*, and dropped from the model). The very strong covariate effect indicates that post-treatment and pre-treatment reaction times are highly correlated among Subjects and Treatments; whereas, the Treatment by Covariate interaction term indicates heterogeneity among treatments in the slopes of these relationships. A post hoc examination of these trends revealed that the two treatments that include meditation, M and SD+M, have shorter Post-Treatment PVT Reaction Times (*F_1,27 _= 7.152, p < 0.02*) and a shallower regression slope (*F_1,27 _= 12.610, p < 0.002*) than the two treatments that do not include meditation, C and N (Figure 2). Separate Analysis of Covariance for C versus N treatments, and for the M versus SD+M treatments indicates no significant effects of Treatment, Subject, Treatment by Covariate interaction, or Treatment by Subject interaction for either grouping.

In our second experiment, our response variable was average sleep duration, which was analyzed with a one-way ANOVA with two treatments: Meditators versus Non-meditators.

All research protocols were reviewed and approved by the University of Kentucky's - Institutional Review Board. All protocols and ethical directives were strictly adhered to including informed consent from all study participants.

## Results

In the first study, we asked whether meditation might impact PVT performance, relative to pre-test conditions and our other treatment groups. In this initial group, we trained 10 students in simple breathing-focused meditation, and asked them to meditate for 40 minutes. PVT performance was assessed just prior to the meditation period, 10 minutes following meditation, and again one hour later. Each subject was tested on two different days with meditation treatment (M), two days with a 40 minute nap (N), and two days with 40 minutes of relaxed eyes-open activity (C). The six days of testing on each subject were spaced over multiple weeks, and each day's activity randomly assigned, unknown to the subject in advance. Later, we examined whether meditation improves PVT performance after 32 hours of sleep deprivation for the same set of subjects, which was done once for each subject.

Our response variable was change in PVT reaction time (pre-treatment - post-treatment). Because we found no effect of Day in our C, N and M treatments (Day: *F_1,47 _= 0.691, p = 0.410*), we averaged the two treatment days for our pre- and post-treatment reaction times for the C, N and M treatments, and used the single day of data following sleep deprivation (four treatments in total).

In all 10 subjects, performance on the PVT improved (faster reaction times) following meditation (*F_1,9 _= 102.454, p << 0.00001*, Figures 1 and 2, Table 1). See the methods section for a more detailed statistical analysis. The direction and amplitude of this trend was congruent for 9 out of the 10 subjects on each of the two days. One hour later, many subjects return to baseline performance with no significant differences relative to control days (data not shown).

In contrast to meditation, the reaction times slowed for 9 out of 10 subjects following a nap (*F_1,9 _= 26.375, p < 0.001*, Figures 1 and 2, Table 1). This trend was congruent on both nap days for 9 out of 10 subjects. The slower times following a nap were probably due to sleep inertia as shown previously[18]. One hour later, reaction times improved, but were still below baseline (data not shown), again consistent with some other studies on the dissipation of sleep inertia[19]. Surprisingly, even the control activities showed a clear slowing on the PVT (*F_1,9 _= 28.295, p =< 0.0005*; Figure 1), perhaps due to a mild circadian decline in performance during the late afternoon (the "mid-afternoon dip"). As is common in college students, our subjects had relatively late bed-times (median of 1:00 am) and wake-times (median 8:30 am) and thus our 4:00 pm post-treatment time may have coincided with their nadir in performance. In light of the results following naps and control activities, the improvement post-meditation appears even greater, since the improvement occurs against the normal decline.

To determine if this post-meditative effect could improve performance under conditions of a large sleep debt, we next challenged each subject with a full night of sleep deprivation. As expected, after approximately 32 hours with no sleep, the subjects had slower baseline PVT measurements (289 ms versus 255 ms; *F_1,56 _= 21.959, p << 0.0001*), and against this lower baseline, the enhancement with meditation was again significant (*F_1,9 _= 15.210, p < 0.005*, Figures 1 and 2, Table 1). In contrast to the initial testing when subjects were well rested, we observed a number of lapses being committed by subjects in this experiment, which were extremely rare in the other treatments. A lapse was defined as an RT of >500 ms or an erroneous response. The number of lapses post-meditation declined both for individuals and as a group (*F_1,7 _= 4.85, p < 0.05, 1-tailed test*), further supporting a global improvement in cognitive and psychomotor alerting responses following meditation.

**Figure 3:**
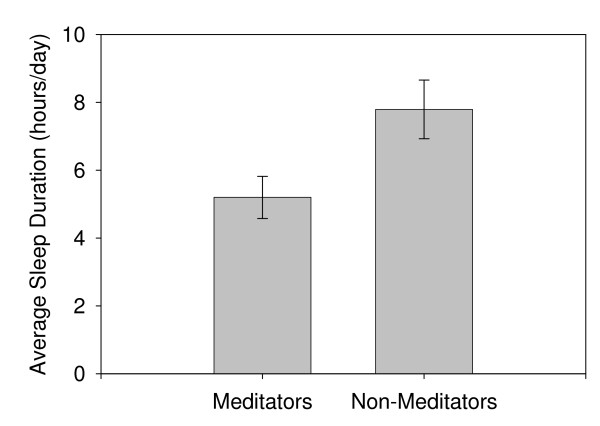
**Average sleep duration for long-term meditators versus non-meditators in India**. Meditators had significantly shorter sleep durations than non-meditators (5.2 versus 7.8 hours per day; *F_1,28 _= 54.183, p <<0.00001*). Error bars denote one standard error.

This experiment suggests that meditation serves a performance-enhancing and perhaps restorative role even in novice meditators. To address this possible restorative role over longer durations, we conducted an initial study on seven long-term "expert" meditators in India, who typically spent 2-3 hrs/day in meditation, versus 23 age- and sex-matched non-meditators as controls. These subjects had sufficient amounts of daily meditation time such that it might produce a noticeable decrease in total sleep time if meditation can actually replace a portion of sleep, or compensate in some other way. As a first step to address this question, we used actigraphy[20], EEG, and sleep journals to assess sleep, wake, and meditation times, and found a generally shorter sleep time in these subjects (Table 2, Figure 3) relative to our control subjects (5.2 versus 7.8 hours per day; *F_1,28 _= 54.183, p <<0.00001*), and also compared to published norms[21]. In addition, there was no evidence of sleepiness on the PVT when it was run multiple times in a subset of these subjects compared to subjects with more typical sleep times (7-8 hours/night) (data not shown). A subset of meditators also underwent Multiple Sleep Latency Tests, to look for day-time sleepiness, and did not display any evidence of sleep debt (mean sleep latency being 18.25 minutes, above population norms). Some subjects were also monitored by EEG, video, and/or direct observation during bouts of meditation to confirm that very little (if any) sleep occurred during the meditation bouts. EEG showed very little sleep of any stage, and video and direct observations showed no signs of head droop or other postural changes indicative of sleep.

**Table 2:** Daily sleep and meditation amounts in meditators vs. controls

Average Sleep Time -- hours/day (average meditation times in parentheses - hours/day)	
**Meditators**	**Non-Meditators**

5.10 (2.50)	7.36
6.00 (2.50)	8.07
4.00 (3.50)	6.77
5.50 (2.20)	8.13
5.50 (2.20)	7.36
5.25 (1.50)	8.11
5.00 (1.75)	6.52
	7.14
	7.57
	9.48
	8.37
	7.75
	8.00
	7.50
	9.41
	8.36
	6.00
	7.04
	8.00
	8.17
	7.13
	9.19
	7.77

## Discussion

A wide variety of claims for different types of meditation have been made over many centuries. Among these claims are improvement in many types of performance and alterations in sleep and EEG patterns that have received considerable, but sometimes mixed, scientific support over the past few decades [3,9,22-25]. Our results demonstrate that a forty minute bout of meditation produces a short-term improvement in PVT performance in novice meditators. Previous studies on meditation and reaction time have focused largely on Transcendental Meditation (TM) that typically involve 20 minute bouts of meditation focused on a Mantra that is claimed to be specifically suited to each practitioner. In some studies, practitioners of TM have faster baseline reaction times, consistent with studies showing improved reaction times in novice subjects undergoing TM training over many days [26,27]. These subjects did not show immediate pre-test to post-test improvement as we demonstrate here. Similarly, in studies of Buddhist mindfulness meditation, reaction times improved over many weeks of practice, but since testing was done shortly after meditation periods [5], it is also possible these subjects benefited from the short-term improvements we find in our present study. It appears that some changes associated with meditation occur with very little training, while others may take many years of practice. For example, with EEG patterns, increases in alpha and theta power in the EEG spectrum occur rapidly with almost any eyes-closed relaxation [1,3], while changes in gamma wave coherence are most remarkable in very experienced Tibetan Buddhist meditators during meditation bouts [23]. Interestingly, these latter changes in gamma activity persist to a lesser degree during non-meditation periods suggesting long-term changes in brain activity in these experienced meditators [23]. This kind of more intense meditation training also appears critical to improvements in attentional tasks [28].

In contrast, the improvement we see in novice meditators may be related to the increase in alpha (8-12 Hz) or theta (5-7 Hz) waves and the increased feelings of relaxation that may decrease distraction on the subsequent PVT, and thus improve performance. In addition, the increase in neuronal synchronization may directly improve the performance of neuronal systems needed for the PVT, perhaps by some kind of "re-setting" to baseline conditions. The role of neuronal synchronization in the cortex reflected by increased amplitude in the EEG for any frequency band is unclear, but certainly the very strong correlation between time spent awake and the subsequent EEG delta power (0.5-4 Hz) during non-REM sleep [29] suggest the possibility that synchronization at delta frequencies during non-REM sleep play some kind of role in neuronal restoration or augmentation. If so, it seems plausible that increased alpha or theta power might be restorative in some manner as well, perhaps in ways similar to non-REM sleep. It has been suggested that subjective reports of restfulness following meditation may reflect that meditation is a sleep-like state, or that substantial sleep occurs during bouts of meditation [30], but this is not supported by the overwhelming majority of EEG studies (see reference 3 for review), nor did we see any evidence of sleep in our subjects during meditation periods.

Based on previous reports of sleep inertia (reviewed in reference [15]), we expected to see a decline in PVT performance following a nap, but were somewhat surprised by the magnitude of this decline (Figure 1). This substantial decline may be due to the duration of our naps that probably allowed at least some subjects to reach deep slow-wave sleep (previously called stage 3 and 4, and now simply N3) that has been associated with increased sleep inertia following arousal [18]. Most of our subjects appeared to fall asleep within ten minutes and thus had 30 minutes of sustained sleep time. The decline in performance following control activities is less clear, but may be due to multiple factors including a continuing decline in alertness at this time of day (3:00-4:00 pm) that represented roughly the mid-point of their 16-17 hour wake period. In addition, although we excluded subjects with high caffeine usage, our subjects were "typical" adults with caffeine intake estimated on the order of 100-200 mg/day. Since we allowed no coffee, tea, soda, or other high caffeine source throughout the afternoon period on each experimental day, it is also possible our subjects were experiencing mild caffeine withdraw or simply missing their usual afternoon caffeine boost that was more severe at 4:00 than 3:00. It is also possible their motivation lapsed slightly during this period for other reasons. In any case, the decline in performance under control conditions suggests that the improved performance following meditation may be closer to 14% (relative to their no meditation control days) than the roughly 7% improvement relative to pre-test conditions (Figure 1, Table 1). In addition, this clearly distinguishes the meditative state from the sleep state, despite the fact that both meditation and sleep (at least for stages 3 and 4) display increased cortical neuronal synchronization [3,7] as seen on EEGs.

Although the frequencies and generation of neuronal synchronization are different in meditation and sleep, the increase during meditation coupled with claims that meditation is restorative, led us to investigate the related question of whether this practice might be able to replace a portion of sleep. While the results presented here are preliminary, they support the possibility of some restoration. All seven Yogis that we studied have sleep times below both the control population we assessed and with published norms from multiple different ethnic groups [21], and all had substantial periods of meditation each day approximately equal to the "lost" sleep. Anecdotal claims that some Yogis do not sleep at all or only two hours/night are probably exaggerated, as are many sleep claims, but a one to one pay-off of sleep debt through meditation appears possible for a portion of sleep. When we challenged Yogis with a total night of sleep deprivation, they were generally too tired to meditate successfully, further discounting the idea that extensive meditation could totally or largely replace sleep. An alternative explanation of our results could be that meditators have deeper or more intense sleep, and thus may be able to achieve the restorative benefits of sleep in less time.

## Limitations

A number of limitations may be relevant to these studies. First, the subjects in the primary study assessing PVT changes were all college age students and novice meditators. Therefore, these results may not extend to other populations that differ in age, meditation experience or skill, type of meditation, education, or many other variables. However, a pilot study on a couple of our experienced meditators and a couple age-matched controls from the second study (age: late thirties to early forties) suggested similar results (data not shown), and the two experienced meditators appeared to maintain their performance improvement into the second hour (not shown again, due to extremely small sample sizes). Even our larger sample size in the primary experiment (n = 10) is admittedly quite small. Nonetheless, power analyses supported this sample size as sufficient using a within subject design, and with the magnitude of improvements we suspected based on the first few subjects. Naturally, a larger sample size would likely have found additional differences between treatment groups or for additional variables. For example, meditation, even in the novice group, appeared to help maintain PVT performance across the entire 10 minute test periods (in the first post-test but not the one in the second hour), whereas performance declined following nap or control periods within these single 10 minute tests. However, we lacked sufficient power to properly test this effect, and it was therefore not included in the results.

Another limitation is the possibility that subjects expected to do better following meditation, either from their own beliefs, or conveyed unconsciously by the investigators. This is a difficult problem in studies of meditation and many other treatments, since double-blind studies are not possible (subjects know which "treatment" they are getting). While we cannot eliminate this possibility in the present study, we think it is unlikely for two reasons. First, several subjects told us they thought that both meditation and the nap would improve their performance, since they were unaware of sleep inertia effects. Clearly their "expectation" following the naps did not improve their performance substantially (since they consistently did worse than their pre-test). Second, the PVT is a very basic test of reaction time and vigilance that has no learning curve and would be difficult to consciously or unconsciously under-perform in order to later out-perform one's results following meditation. Further, our subjects were young and highly motivated, and we encouraged them to perform their best on all tests. In summary, we believe we would have seen evidence if our results were primarily due to the subjects' or the investigators' expectancies. These issues will continue to be important as we try to expand our studies from PVT performance to cognitive performance or other functions. In the latter study on Yogis and controls in India, it is possible that Yogis wished to exaggerate the benefits of yoga and the reduction in sleep it allowed, but it seems unlikely to us that they would have maintained such limited sleep for the full duration of these studies. In addition, it is for this reason we insisted on at least 15 days of actigraphy data on each Yogi and not simple sleep journals that are much more easily biased.

It may be of interest to note that our seven Yogis all slept between four and six hours a day. Perhaps, there is a basic core amount of sleep that is critical to all mammals (and perhaps all or most non-mammals too) and cannot be replaced by anything but sleep. Sleep time in mammals varies from about four to nineteen hours [31,32], potentially supporting a view that some portion of sleep in longer sleeping mammals is adaptive and not critical for basic brain functions. Some researchers have suggested that humans have a core need of four to six hours of sleep per day [33]. However, considerable data have shown that chronic restriction to this amount of sleep in most humans results in poor performance and the accumulation of substantial sleep debt [16]. While there is still some debate regarding these issues [34,35], there is general consensus that the majority of adults need about 7 hours or more per night for optimal or near-optimal function without accumulating a sleep debt. Yet, our limited data to date suggest that the Yogis in this study are near or exceed the optimal range on PVT and MSLT with less than 7 hours of sleep, and may be replacing one to three hours of sleep with meditation (despite no signs of sleep during these meditation bouts). Planned EEG studies in meditators may provide supporting data if we find that high alpha or other synchronous firing during meditation reduces the EEG delta power during subsequent sleep (or in the alternative explanation above, might increase EEG delta power as the Yogis sleep more intensely). It is also possible that meditation might be able to do whatever it is that sleep does, by a different but overlapping process. In either case, if meditation is restorative in a manner similar to sleep, it might benefit individuals with excessive daytime sleepiness due to sleep disorders or to lifestyle factors. Not only might these meditation bouts reduce accumulating sleep debt, but individuals may also benefit from the short-term performance improvements noted above, without the problems of sleep inertia that occur with longer naps. Lastly, so-called "power naps" of 10-15 minutes duration often involve no actual sleep, and may therefore provide a period of predominant alpha waves more similar to meditation than sleep.

## Conclusions

Meditation appears to provide at least a short term improvement in reaction time performance, and may also provide a longer term reduction in sleep need roughly equal to the time spent in meditation.

## Competing interests

The authors declare that they have no competing interests.

## Authors' contributions

PK carried out all experiments with assistance from JP. PK also contributed to all other aspects of the project including the design and implementation of all studies, data analyses, manuscript preparation, etc. RS performed all statistical analyses and contributed to the design of experiments, interpretation of data, and writing the manuscript. BO conceived of the project and design of the studies, provided funding, interpreted data, and directed all other efforts. All authors read and approved the final manuscript.
